# Tracing retinal vessel trees by transductive inference

**DOI:** 10.1186/1471-2105-15-20

**Published:** 2014-01-18

**Authors:** Jaydeep De, Huiqi Li, Li Cheng

**Affiliations:** 1 Bioinformatics Institute, A*STAR, Singapore, Singapore; 2 School of Computer Engineering, Nanyang Technological University, Singapore, Singapore; 3, Beijing Institute of Technology, Beijing, China; 4 School of Computing, National University of Singapore, Singapore, Singapore

## Abstract

**Background:**

Structural study of retinal blood vessels provides an early indication of diseases such as diabetic retinopathy, glaucoma, and hypertensive retinopathy. These studies require accurate tracing of retinal vessel tree structure from fundus images in an automated manner. However, the existing work encounters great difficulties when dealing with the *crossover* issue commonly-seen in vessel networks.

**Results:**

In this paper, we consider a novel graph-based approach to address this tracing with crossover problem: After initial steps of segmentation and skeleton extraction, its graph representation can be established, where each segment in the skeleton map becomes a node, and a direct contact between two adjacent segments is translated to an undirected edge of the two corresponding nodes. The segments in the skeleton map touching the optical disk area are considered as root nodes. This determines the number of trees to-be-found in the vessel network, which is always equal to the number of root nodes. Based on this undirected graph representation, the tracing problem is further connected to the well-studied transductive inference in machine learning, where the goal becomes that of properly propagating the tree labels from those known root nodes to the rest of the graph, such that the graph is partitioned into disjoint sub-graphs, or equivalently, each of the trees is traced and separated from the rest of the vessel network. This connection enables us to address the tracing problem by exploiting established development in transductive inference. Empirical experiments on public available fundus image datasets demonstrate the applicability of our approach.

**Conclusions:**

We provide a novel and systematic approach to trace retinal vessel trees with the present of crossovers by solving a transductive learning problem on induced undirected graphs.

## Background

Topological and geometrical properties of retinal blood vessels from fundus images can provide valuable clinical information in diagnosing diseases. In particular, vascular anomaly in retina is one of the clinical manifestations of retinal diseases such as diabetic retinopathy, glaucoma, and hypertensive retinopathy. Take diabetic retinopathy as an example, it is a leading cause of blindness in the working-age population of most developed countries. Diabetic retinopathy is the result of progressive damage to the network of tiny blood vessels that supply blood to the retina. It is classified into two major groups in clinics according to the severity of the disease: non-proliferative and proliferative. Proliferative diabetic retinopathy is characterized by the formation of new-formed vessels in the retina, while non-proliferative diabetic retinopathy refers to the absence of abnormal new blood vessels [1]. The description of blood vessel tree structure is therefore essential in clinical diagnosis of eye diseases such as diabetic retinopathy. Unfortunately, commercial softwares still largely rely on manual tracing of the blood vessel trees. This is tedious and time-consuming due to the highly variable structure of these retinal vessels, and is not sustainable for high-throughput analysis in clinical setting.

Existing efforts in retinal vessel analysis can be roughly categorized into two groups, namely, segmentation-based and tracking-based. The segmentation-based methods often use pixel classification [2-9] to produce a binary segmentation, where a pixel is classified into vessel or non-vessel. Ricci et al. [10] work with orthogonal line operators and support vector machine to perform pixelwise segmentation. Mendonca et al. [2] use four directional differential operators to detect the vessel centerlines, which are then engaged for morphologically reconstructing the vessels. An unsupervised curvature based vessel segmentation method is proposed by Garg et al. [3]. Meanwhile, a deformable contour model is adopted by Espona et al. [4], by incorporating the snake method with domain specific knowledge such as the topological properties of blood vessels. Soares et al. [6] adopt eighteen dimensional Gabor response feature to train two Gaussian mixture models (GMMs), which are further employed to produce a binary probability map for a test image. The tracking-based methods [11-21], on the other hand, usually start with a seed and track the intended vessel based on local intensity or texture information. The authors of [12] divide the image into non-overlapping grid and considered each grid separately for seed finding, which are followed by the tracking procedure to uncover the vessel network structure. In a series of research efforts [18-21], the authors extract *tubularity* measure of all image pixels and connect those pixels having high tubularity measure values. Then, an optimal set of trees over these tubular pixels are selected by minimizing a global objective function with prior geometric constraints, such as orientation and width of the vessel structure. In a recent work [22], the tracking problem is formulated as a constrained optimization problem based on vessel orientation and topology, and a candidate enumeration algorithm is devised for the proposed optimization problem, which prunes the search space by maintaining a lower bound on the objective function. Meanwhile, there are also research efforts from the related neural tracing community that utilize graph based methods and achieve promising results, such as the all path pruning methods of [23,24].

It has been observed that segmentation-based methods tend to produce many disconnected and isolated segments [10], which are less favourable towards retaining the important topological properties of vessel networks [25]. Vessel tracking methods, on the other hand, often preserve the connectivity structure of vessel segments. Nonetheless they encounter great difficulties with the occurrence of *crossover*[15] at the junction points. Current methods often fail to trace properly, as it is nontrivial to predict whether the vessel segments touching at a junction point belong to one tree, or two and more trees, and for the later case, to which tree each segment belongs. In this paper, we dedicate our attention to addressing this bottleneck issue, which is referred to as the *crossover* issue. One important observation is that local and global contextual information is crucial to resolve the crossover issue. For example, at a junction point, it is very helpful to go beyond the current vessel brunch and examine the angular properties of the other vessel brunches of the junction. These information is unfortunately ignored by current tracing methods. This observation inspires us to consider a different tracing approach that can take into account both local and global contextual information of the vessel network: After initial steps of pixel-based segmentation and skeleton extraction, a novel *graph representation* is formed, where each segment in the skeleton map becomes a node, and a direct contact between two adjacent segments is translated to an edge of the two corresponding nodes. The segments in the skeleton map touching the optical disk area are considered as the root nodes. The number of trees to-be-found in the the vessel network thus equals the number of root nodes. This graph representation is further simplified using a modified version of segment ordering [26-28]. Based on the graph representation, the tracing problem is further formulated as a transductive inference problem in machine learning, where the goal becomes that of propagating the tree labels from known root nodes to the rest of the graph, such that the graph will be split into disjoint sub-graphs, which corresponds to trees of the vessel network.

The main contributions of this paper are three-fold. First, our approach offers a principled way of addressing the crossover issue. By connecting to the well-established transductive inference in machine learning [29], both local and global contextual information can be explicitly considered. Second, a novel graph representation is proposed, which can be regarded as an equivalent *dual* representation of the original vessel network, and is essential for establishing the machine learning connection. Third, the graph representation is simplified using a modified version of segment ordering which is meaningful for tracing purpose. We expect the graph representation, and the transductive-inference connection can open the door to some insightful understanding of the characteristics of crossover sections in vessel networks.

## Methods

**Figure 1:**
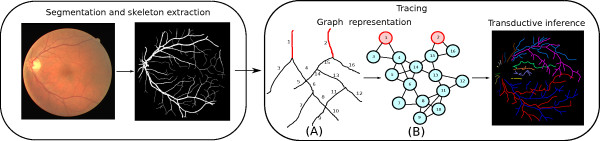
**Flowchart of our approach.** Overview of the proposed tracing pipeline (Best view in color).

Figure 1 provides a flowchart description of our approach, which contains two main steps: The segmentation step focuses on faithfully retaining the small branches as well as the connectivity between neighbouring branches. The tracing step, which contains our main contributions, turns the tracing problem into graph-based transductive inference, and the final tracing results are obtained by exploiting the well-studied transductive inference tools in machine learning.

### Segmentation

The goal of the segmentation step in our context is to extract vessel skeletons while maintaining their structural connectivity, as well as the corresponding point-wise thickness along the skeletons – based on which the retinal vessels can be faithfully reconstructed. This differs notably from the usual aim of most existing segmentation work, where the emphasis is to achieve a high classification accuracy. As the number of vessel pixels are much fewer comparing to the number of background pixels, often a high accuracy is achieved by missing many vessel pixels, a situation we try to avoid. In fact, our goal can be better described as *segmentation with a high recall*. In other words, it is critical for us to retain the vessel pixels that keep the local vein and artery branches from being broken or entirely missing. To achieve this, based on two existing methods [6,8], our segmenter is formed by merging the results in a sequential manner, while emphasizing on retaining the right network connectivity. Since our main focus is the tracing step, we discuss here only the quantitative analysis of the segmentation results, and leave the detailed description of our segmentation step to Appendix.

**Figure 2:**
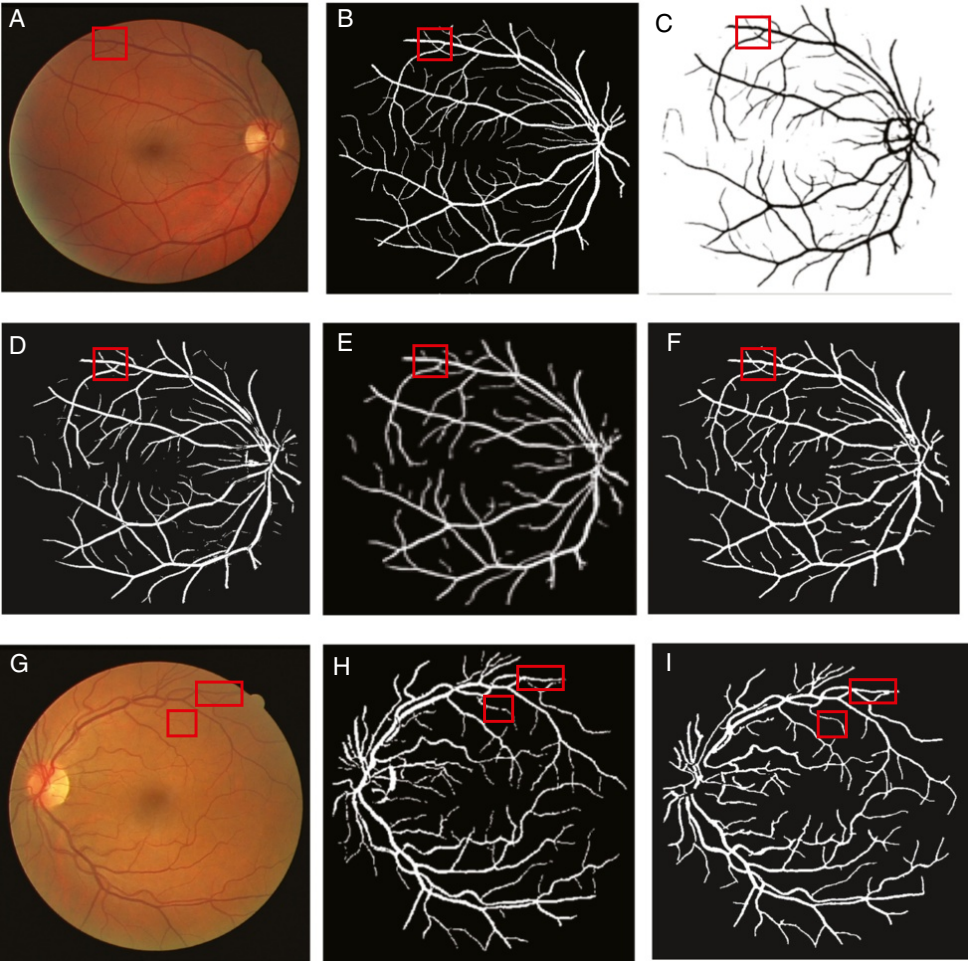
**Visual comparison of the segmentation result on an exemplar fundus image.(A)** Original image (DRIVE-Test-Image-19), and its ground-truth segmentation in **(B)**. **(C)** Segmentation result of [10]. **(D)** Segmentation result of [6]**(E)** Segmentation result of [7]. **(F)** Segmentation result of our method. **(G)** Original image (DRIVE-Test-Image-01). **(H)** Segmentation result of [5]. **(I)** Segmentation result of our method. If we pay close attention to the boxed regions, we can see that our segmentation step does a much better job at retaining the connectivity of the skeleton.

To evaluate the segmentation performance, Table 1 compares our results to the state-of-the-art methods at the popular DRIVE dataset, while our segmenter excels in picking up most vessel pixels (the recall column) – crucial for maintaining the connectivity of segmented parts, it also performs reasonably well in keeping only a small fraction of false alarms (the precision column) and thus a very competitive accuracy score – comparable with the leading methods. Visually our algorithm also performs significantly better than the existing ones in term of preserving the connectivity among segments, which is crucial for tracing, as displayed in Figure 2. Figure 2(F) and (I) are two examples of our segmentation results. Figure 2(C-E) and (H) are examples of other state-of-art methods. The areas shown in red boxes are the areas where our segmentation algorithm works better than others and it retains the connectivity of the vessel network.

**Table 1:** Comparison of vessel segmentation performance in DRIVE

**Method**	**Precision**	**Recall**	**F1**	**Acc.**
Ricci [10]	-	-	-	.9563
Mendonca [2]	.7315	-	-	.9463
Peter Bankhead [8]	.7027	.7177	.7101	.9371
Garg [3]	-	-	-	.9361
Espona [4]	.7436	-	-	.9352
Martinez-Perez [5]	.7246	-	-	.9344
Diego Marin [7]	.8433	.7067	.7690	.9452
Soares et al. [6]	.6943	.7425	.7176	.9466
Our method	.7602	.8336	.7952	.9429

At the final stage of the segmentation step, the binary segmentation result is converted into a skeleton map (of one pixel thickness) by standard medial-axis transform. Meanwhile the optical disk region is identified and removed by applying a simple smoothing and thresholding step.

### Tracing

**Figure 3:**
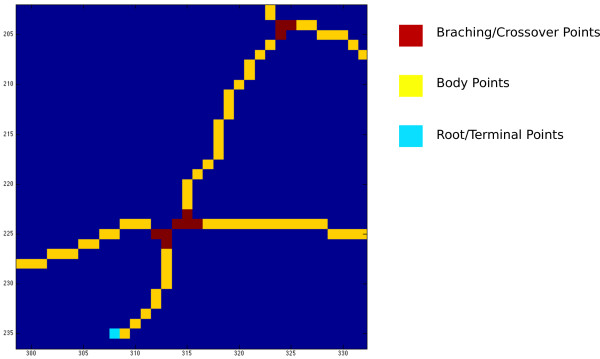
**Notation on the skeleton pixels.** Skeleton pixels are classified into the following three types: *Body* points – Pixels having 2 neighbors; *Branching* points – Pixels having 3 or more neighbors; *Terminal* points – Pixels having 1 neighbor, being either root or end pixels. A *segment* is thus defined as a continuous subset of skeleton pixels starts with a terminal/branching point, and ends with a branching/terminal point.

Let us start by settling down few definitions. In the skeleton map, pixels can be partitioned into the following three categories (also illustrated in Figure 3): 

• *Body* Points: Pixels having two neighbours.

• *Terminal* Points: Pixels having one neighbour.

• *Branching* Points: Pixels having three or more neighbours.

In particular, the terminal points residing inside the removed optical disk area are called the *root* points, and the remaining terminal points are *end* points. A *segment* is thus defined as a continuous subset of skeleton pixels starts with either a root or a branching point, and ends with a branching or an end point.

It is commonly assumed that for tracing, we always start from the optical disk where the root points of the retinal vessel skeleton are present. As a result, the retinal vessels can always be separated into a *disjoint* set of rooted trees, with each tree possessing a unique label stemming from its root point (and thus *the* segment it resides in). This is illustrated as the red-coloured segments of the vessel skeleton in Figure 1 (A). Clearly the number of trees is always known a priori at this stage.

#### ***From skeleton map to graph representation***

The skeleton map of the segmented image is converted into an undirected graph, *G*=(*V*,*E*), where the nodes *V* are the vessel segments of the image and there is an edge between two nodes if the corresponding vessel segments are connected in the image. Figure 1 (A) and (B) provide an illustrative example of transforming a small fraction of a skeleton. The segments containing the root points are each regarded as the root node of a distinct tree (Figure 1-A, B). The number of possible labels per node equals the number of root nodes in the graph. As the label for the root nodes are known, they are regarded as labelled nodes (nodes in red color).

#### ***Graph simplification based on segment ordering***

**Figure 4:**
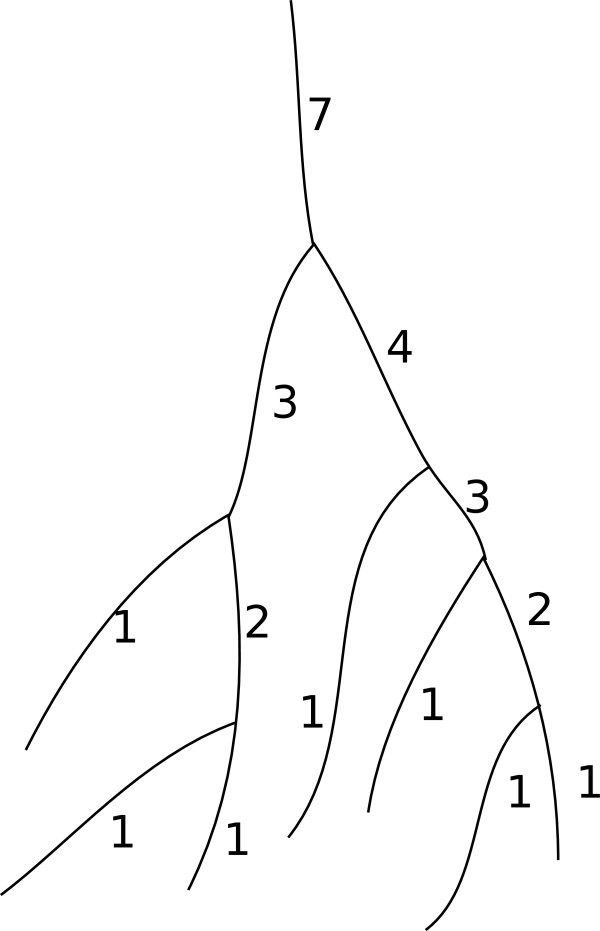
**Shreve’s ordering.** The vessel segments are ordered hierarchically. The ordering starts from the leaf segments which are assigned an order of 1. When two segments of order *μ*_1_ and *μ*_2_ meets then the resulting segments are given an order of *μ*_1_+*μ*_2_.

The graph that we have constructed so far contains redundant edges that can be further simplified based on segment ordering. Segment ordering refers to the graph-theoretical algorithms (such as [26-28]) aiming to order the segments following the underlining network structure. As displayed in Figure 4, it works by first assigning lowest integer values to the terminal segments (segments containing the terminal points), and assigning integer values in a non-decreasing manner following the network structure as we move up along the path from the terminal (leaf) segments towards the root segment. Segment ordering has been applied, for example in the hydrology applications to order the streams from a hierarchy of tributaries [26,27]. In this work we adopt the Shreve’s ordering [28] and propose a modified version of it.

#### ***Shreve’s ordering***

Shreve’s ordering is defined on trees as follows: 1) Each terminal nodes are assigned an order of 1. 2) If two segments of order *μ*_1_ and *μ*_2_ meet then the resulting segment obtains an order of *μ*_1_+*μ*_2_. Figure 4 illustrates an example of Shreve’s ordering. Shreve’s ordering assumes that the network structure is a tree with a single root with no crossover, which is true for river networks. However, we need to take care of crossover situations and multiple roots in our context, with which we propose a modified version of Shreve’s ordering.

#### ***Modified Shreve’s ordering***

**Figure 5:**
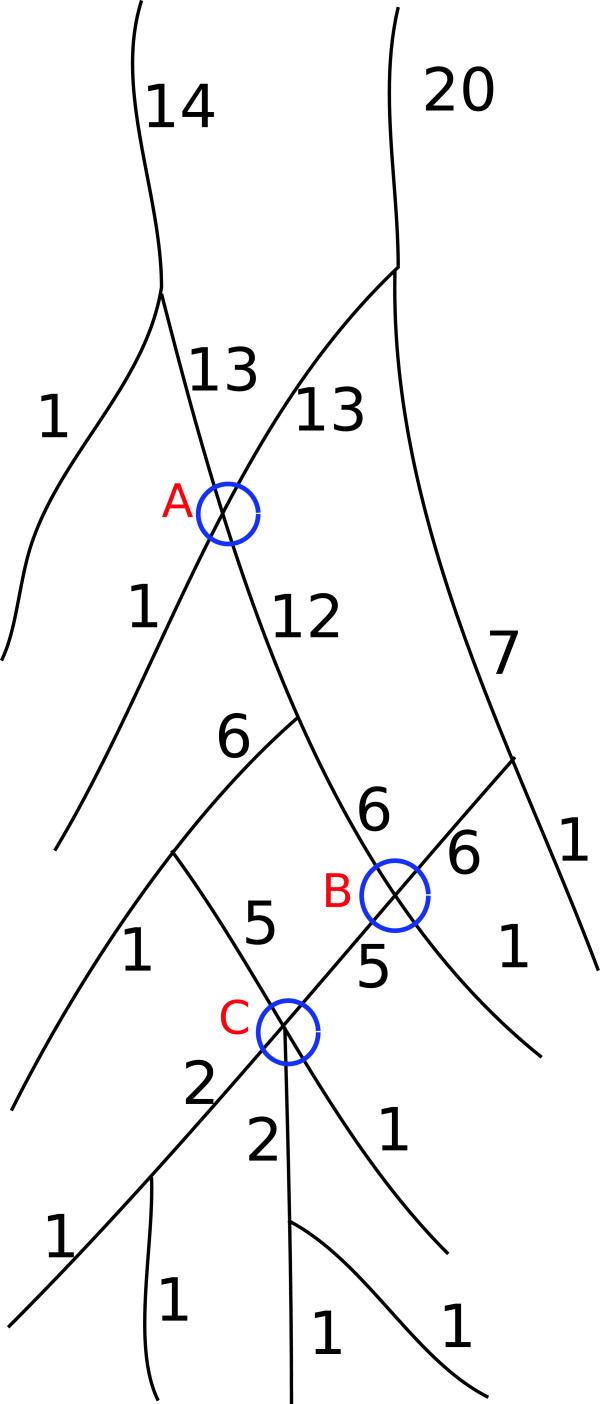
**Modified Shreve’s ordering.** Shreve’s ordering is modified to handle crossovers like A, B and C. For 4-cliques like A and B, when two segments with order *μ*_1_ and *μ*_2_ meets then the resulting segments are ordered as *μ*_1_+*μ*_2_. Similarly for 5-clique like C, when three segments with order *μ*_1_, *μ*_2_ and *μ*_3_ meets then the resulting segments are ordered as *μ*_1_+*μ*_2_+*μ*_3_.

Our modified version of the Shreve’s ordering algorithm is defined below. 1) Each terminal nodes are assigned an order of 1. 2) In a 3-clique, if the two incoming segments has order of *μ*_1_ and *μ*_2_, then the the third (outgoing) segment will be ordered as *μ*_1_+*μ*_2_. 3) In a 4-clique, if the two incoming segments has order of *μ*_1_ and *μ*_2_, then the other two (outgoing) segments will be ordered as *μ*_1_+*μ*_2_. 4) In a 5-clique, if the three incoming segments has order of *μ*_1_, *μ*_2_ and *μ*_3_, then the remaining two outgoing segments will be ordered as *μ*_1_+*μ*_2_+*μ*_3_. 5) In a 6-clique, if the four segments has order of *μ*_1_, *μ*_2_, *μ*_3_ and *μ*_4_, then the rest two outgoing segments will be ordered as *μ*_1_+*μ*_2_+*μ*_3_+*μ*_4_. In Figure 5, the blue circle marked as A and B are two examples of 4-cliques and C is an example of 5-clique. Note by incoming and outgoing here we refer to the directional paths each starts from a terminal (leaf) segment and ends at its root segment.

**Figure 6:**
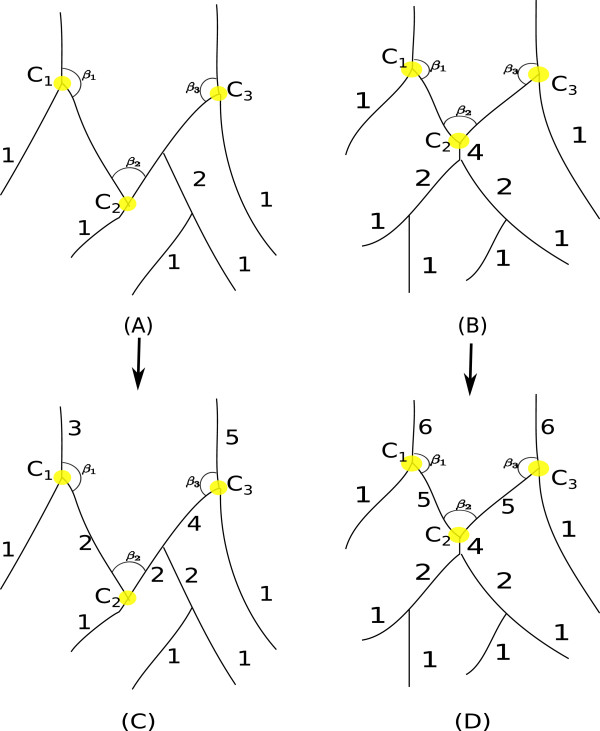
**Another modification in Shreve’s ordering.** Shreve’s ordering is modified further to handle erroneous 3-cliques like *C*_2_ in sub-figure **(A)** and **(B)**. The *C*_2_ in sub-figure **(A)** is caused by one segment terminating on another segment whereas the *C*_2_ is sub-figure **(B)** is caused by wrong skeletonization of a 4-clique. In both the situations, segments cannot be ordered further as for a 3-clique at least two segments should be already ordered to assign an order to the third segment. This deadlock is overcome by measuring the angles between other segments for all those 3-cliques where only one segment is already ordered (let’s assume the order is *μ*). Among all these cliques we assign an order of *μ*+1 (for two unordered segments) to the clique which has smallest angle. The resulting orders are shown in sub-figure **(C)** and **(D)**.

As shown in Figure 6, there are two issues when dealing with the topological connections of 3-cliques, where Shreve’s ordering algorithm will not proceed properly. The first issue as presented in subplot (A), illustrates the deadlock scenario when running from bottom up, the method of Shreve’s ordering will stop at *C*_2_ (shown in yellow), due to the context of only one incoming segment and two outgoing ones. The second issue is very similar but involves a small spurious segment (i. e. segment 4), as in subplot (B). One key observation here is the angle *C*_2_ in both scenarios is usually an acute angle, while the consecutive angles *C*_1_ and *C*_3_ are usually obtuse angles. By exploiting this fact in both scenarios, we resume the ordering process by searching for the smallest angle along the ordering front line and order the two outgoing segments by +1, as shown in subplots (C) and (D), respectively.

#### ***Graph simplification***

**Figure 7:**
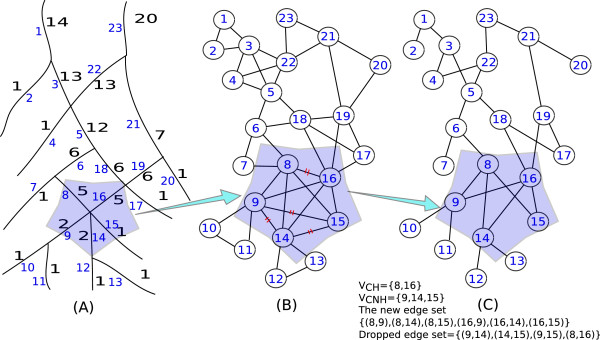
**A graph simplification example.(A)** A skeleton. The digits in black denotes segment orders whereas the digits in blue denotes segment indexes. **(B)** An unsimplified graph. **(C)** A simplified graph. The highlighted segments are particular examples of graph simplification.

Based on segment ordering, now we are able to further simplify the graph. In a clique (3, 4, 5 or 6) there will be one or two nodes which has highest order within the clique. These nodes are hierarchically higher in topological connectivity than the other nodes within that clique. For graph simplification we do the following: 1) We divide a clique *V*_*C*_ = {*v*_1_,*v*_2_,…,*v*_*n*_} into two subsets *V*_*CH*_ = {*v*_1_,*v*_2_,…,*v*_*k*_} and *V*_*CNH*_ = {*v*_*k*+1_,*v*_*k*+2_,…,*v*_*n*_} (i.e. *V*_*C*_ = *V*_*CH*_∪*V*_*CNH*_) where *V*_*H*_ is the set of nodes having highest order within that clique and *V*_*CNH*_ are the rest of the nodes. 2) Now the new edge set will be {(*v*_1_,*v*_*k*+1_),(*v*_1_,*v*_*k*+2_),…,(*v*_*k*_,*v*_*n*_)}. An example of graph simplification is shown in Figure 7, where subplot (B) is the input graph and (C) the corresponding simplified graph.

#### ***Tracing as transductive inference***

Transductive inference was introduced in the mid-70s and has since become popular in machine learning. It is also closely related to semi-supervised learning^1^. Compared to inductive learning, that aims at estimating an unknown function over the entire space, transductive inference focuses on estimating the values of an unknown function on only a few points of interest. This fits precisely into our context. By leveraging this insight, a number of learning algorithms, notably the label propagation algorithm of [30], have been developed to address real-life applications.

Formally, assume there are *n* nodes in our graph representation from vessel skeleton. *Y*_*l*_ = (*y*_1_,*y*_2_,…,*y*_*l*_) denotes the *l* root nodes (red nodes) that are observed, while *Y*_*U*_ = (*y*_*l*+1_,*y*_*l*+2_,…,*y*_*n*_) denotes the rest unobserved nodes (blue nodes). Given this graph representation, the tracing problem can be reformulated as a problem of transductive inference, by propagating the labels from the known root nodes (red nodes) to the rest nodes (blue nodes) in current graph. Clearly it is an easy problem when the subgraphs are isolated from each other (i.e. without crossover in the skeleton map), and becomes increasingly difficult as there are more and more crossovers in the skeleton map. The label propagation algorithm of [30] is adapted to our context as illustrated in Algorithm 1. By starting with the initial guess Y^(0), it is not difficult to show that this algorithm will always converge to Y^(∞)=(1-α)(I-αL)-1Y^(0), and the rate of convergence depends on the eigenvalues of the graph Laplacian [30].

##### **Algorithm 1 Label Propagation (Zhou et al. [**[30]**])**

In practice, the output label Y^(∞)≜Y^(T) is obtained in finite steps when the change of labels ∥Y^(T)-Y^(T-1)∥≤ε, with *ε* ≤ 1*e* - 5. We also empirically fix *α* to 0.9 during our experiments.

#### ***Computing the weight matrix W***

The weight matrix *W* is a real symmetric matrix of size *n* × *n*, which is sometimes referred to as the affinity matrix in graph theory. Clearly *W* is of central importance in our approach as it is assumed to encode sufficient information from the input image data. In this paper, the orientation-based features are proposed as the sufficient statistics toward computing *W* as below: 

• *Segment orientation and angle between segments:* For each skeleton point, the first eigenvector of the Hessian matrix of local image patch determines an orientation. A (usually curved) vessel segment comes with two ends, thus has two local orientations. For each end, its orientation is computed by taking an average of the eigenvector of the last ten skeleton points from this end. It is then used to compute *θ*∈ [ 0,*π*), the angle between two adjacent segments.

We also devise three functions that will be used in constructing *W*. They are

(1) $$\;f_{1}(\theta )=\left\{\begin{array}{c} -\frac{\sin(\theta )}{k}\;\text{if}\;\;\theta \in \;[\;0,\theta_{c})\\ -\frac{\text{sin}(\theta_{c})}{k}\;\text{if}\;\;\theta \in \;[\;\theta_{c},\arccos\left(\frac{-\sin\theta_{c}}{k^{2}}\right))\\ k\;\cos(\theta )\;\text{if}\;\;\theta \in \;[\;\arccos\left(\frac{-\sin\theta_{c}}{k^{2}}\right),\;180\text{°}), \end{array}\right)$$

(2) $$\;f_{2}(\theta )=k\;\text{cos}(\theta ),$$

(3) $$\;f_{3}(\theta )=k+k\;\text{sin}(\theta ),$$

where the parameters *k* and *θ*_*c*_ are experimentally fixed as *k* = 5 and *θ*_*c*_ = 80°.

The diagonal elements of the weight matrix *W* are always zero, *W*_*ii*_ = 0, ∀*i*. The computation of the weight matrix elements *W*_*ij*_ for two different segments *i*≠*j* involves the following rules: 

• *3-Clique:* i.e. there are three adjacent segments in the junction.

• In this scenario we use the following equation:

(4) $$W_{\text{ij}}=\exp(-f_{1}(\theta )).$$

**Figure 8:**
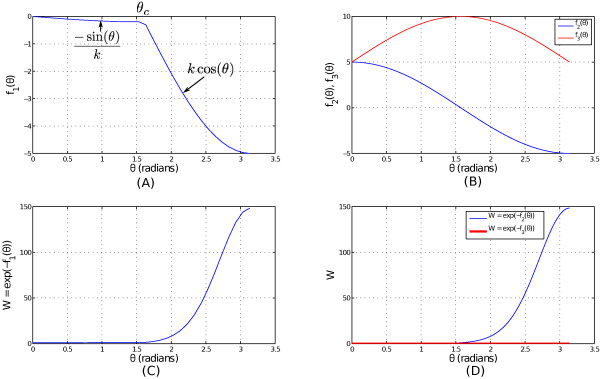
**Influence of*θ* on the values of functions*f***_**1**_**,*f***_**2**_**,*f***_**3**_** and their corresponding*W*.(A)** Variation of *f*_1_(*θ*) with *θ*. **(B)** Variation of *f*_2_(*θ*) and *f*_3_(*θ*) with *θ*. **(C)***W* corresponding to sub-figure **(A)**. **(D)***W* corresponding to sub-figure **(B)**.

**Figure 9:**
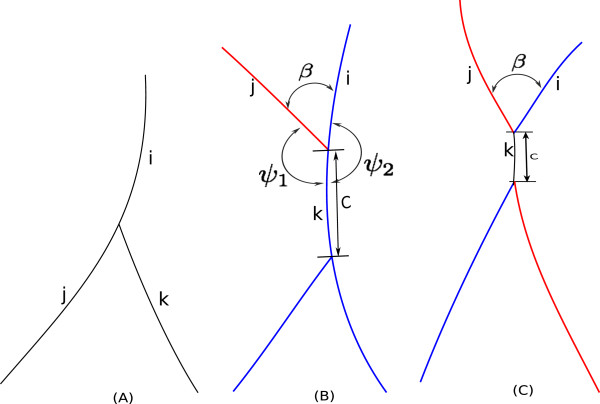
**3-cliques.(A)** A simple 3-clique. **(B)** A 3-clique created by the termination of one segment (red one) on another segment (blue one). **(C)** Two 3-cliques created by the wrong skeletonization of a 4-clique

• The rationale behind choosing this function comes from the intuition that we want to encourage small changes of local curvatures between two connected segments, while punishing those with larger curvature changes. Figure 8(A) and (C) shows the effects of varying *θ* in *f*_1_ and *W*.

• Figure 9 presents three exemplar 3-cliques: subplot (A) (case-A) is the standard branching situation where the segments (marked as *i*,*j* and *k*) belong to the same label. In subplot (B) (case-B), the red segment terminates on a blue segment and creates a 3-clique. Here the segment *i* should have a label different that of segment *j* and *k*. In subplot (C) (case-C), a crossover between the blue and the red branches is converted into two 3-cliques due to error in skeletonization. We differentiate case-A from case-B,C with the help of the segment ordering algorithm explained previously. Case-B and case-C and further differentiated by checking the length of segment *k* (*C* pixels). If *C* ≤ *C*_*critical*_ then we consider it as case-C, otherwise it is case-B. The value of *C*_*critical*_ is experimentally set as *C*_*critical*_ = 10.

• Then we find the orientation difference for two segment pairs (*i*,*k*) and (*j*,*k*). Those two angles are *ψ*_2_ and *ψ*_1_ (shown in Figure 9(B)). Now we calculate the *W*_*ik*_ and *W*_*jk*_ as following: If *ψ*_1_≥*ψ*_2_ then,

(5) $$\begin{array}{lll} W_{\text{jk}} & =\exp(-f_{1}(\theta ))\; & \;\\ W_{\text{ik}} & =\exp(f_{1}(\theta ))\; \end{array}$$

• else

(6) $$\begin{array}{lll} W_{\text{ik}} & =\exp(-f_{1}(\theta ))\; & \;\\ W_{\text{jk}} & =\exp(f_{1}(\theta ))\; \end{array}$$

**Figure 10:**
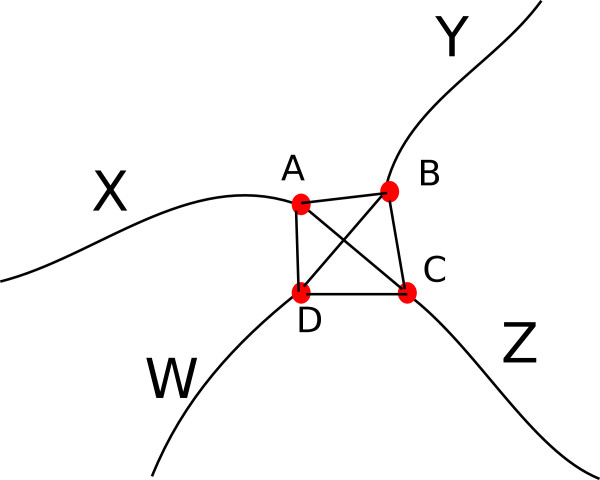
**A 4-clique.** Here we know the (*x*,*y*) coordinates of points *A*,*B*,*C*,*D* in the image plane, so we can find the equation of straight lines $\bar{\text{AC}},\bar{\text{BD}},\bar{\text{AB}},\bar{\text{BC}},\bar{\text{CD}},\bar{\text{AD}}$. We take the pair of straight line equations $\left(\bar{\text{AC}},\bar{\text{BD}}),(\bar{\text{AB}},\bar{\text{CD}}\right)$ and $\left(\bar{\text{AD}},\bar{\text{BC}}\right)$ and find the intersecting points between the pairs. Only one pair $\left(\bar{\text{AC}},\bar{\text{BD}}\right)$ intersect within the convex hull of points (*A*,*B*,*C*,*D*) while others pairs are either parallel or will intersect outside the convex hull. By using this we understand that *A* pairs with *C* and *B* pairs with *D*. Accordingly we assign (*X*,*Z*) (The segments containing points *A* and *C*) and (*W*,*Y*) (The segments containing points *B* and *D*) pair with weight from Equation 7 (Blue curve in Figure 8-D) and for other pairs we assign from Equation 8 (Red curve in Figure 8-D).

• *4-Clique:* i.e. there are four fully-connected segments in the junction.

• As displayed in Figure 10, if *A*,*B*,*C* and *D* are four pixels connecting the four segments *X*, *Y*, *W* and *Z* in a crossover setting, intuitively we can see that only $\bar{\text{AC}}$ and $\bar{\text{BD}}$ line should intersect with each other. The other pairs $\left(\bar{\text{AB}},\bar{\text{CD}}\right)$ and $\left(\bar{\text{AD}},\bar{\text{BC}}\right)$ are not able to intersect within the convex hull of the four points (*A*,*B*,*C*,*D*). Hence from the set of feasible line segments $\left\{(\bar{\text{AB}},\bar{\text{CD}}),(\bar{\text{AD}},\bar{\text{BC}}),(\bar{\text{AC}},\bar{\text{BD}})\right\}$ we can easily identify the $\left(\bar{\text{AC}},\bar{\text{BD}}\right)$ pair that are able to crossover. As a result, higher weight should be assigned to the segment pair (*X*,*Z*) (the segments which contains the points A and C) and (*Y*,*W*) (the segments which contains the points B and D). The subplots of Figure 8(B) and (D) suggest to define the following function form

(7) $$W_{\text{ij}}=\exp(-f_{2}(\theta ))$$

• for these pairs of interest, as well as the function form

(8) $$W_{\text{ij}}=\exp(-f_{3}(\theta ))$$

**Figure 11:**
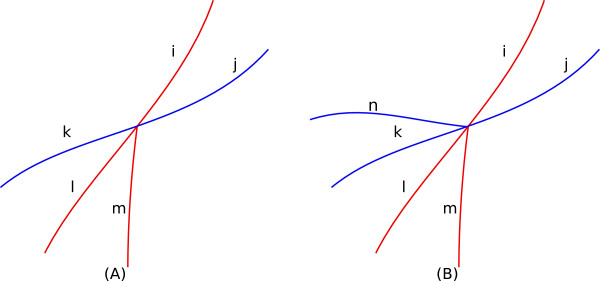
**5- and 6-cliques.(A)** A 5-clique. **(B)** A 6-clique. In both the scenarios weights are assigned using the same function as 3-cliques.

• for the rest less favourable pairs.

• *5/6-Clique:* As presented in Figure 11(A), for 5-clique scenario, one segment (the blue ones) crosses over another segment (the red ones) at the branching point. Here the goal is to divide them in two groups, one having 2 segments (*j* and *k* in subplot (A)) and the other having 3 segments (*i*,*l* and *m*). From segment ordering we know (*i*,*j*) are assigned larger values than the rest, so we already have one member from each group. The rest of the members can be assigned by the usual “smooth curve around the junction point” assumption and employing *f*_1_(*θ*) in the same way as in 3-clique.

• As presented in Figure 11(B) for 6-clique two crossovers happen at the same location. The target here is also to divide them in two groups: one having 3 segments (*i*,*l* and *m*) and the other having 3 segments (*j*,*n* and *k*). Similarly, from the segment ordering we already know that the two nodes with large index values (*i*,*j*), belong to different groups, so we employ *f*_1_(*θ*) in a same way as in 5-clique.

#### ***Removing spurious segments***

**Figure 12:**
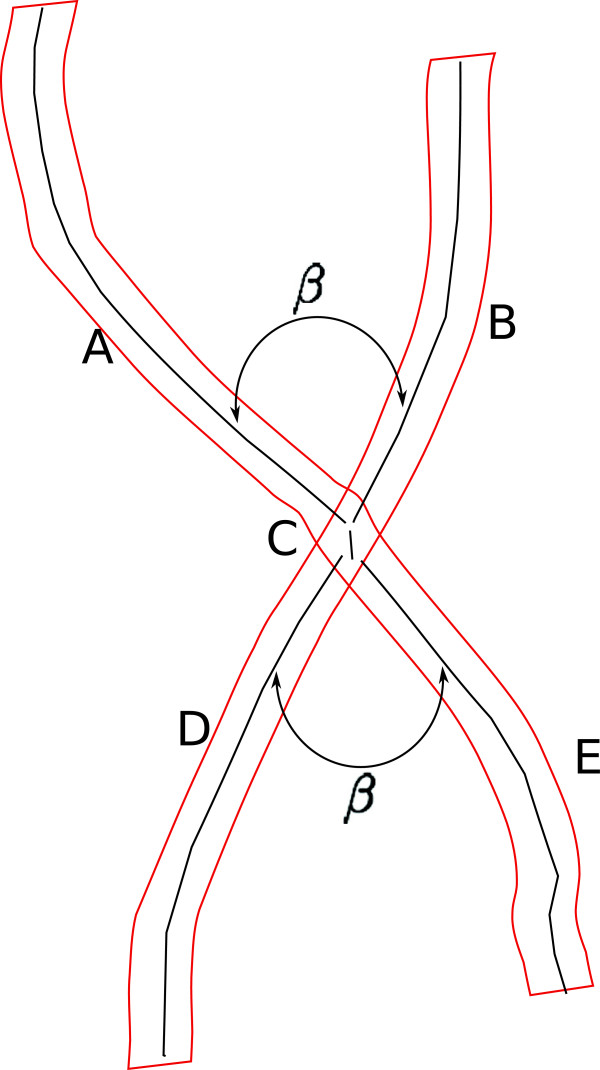
**The introduction of small spurious segments.** An illustrative example of the introduction of a small spurious segment when converting a binary segmentation into its skeleton map.

When converting a binary segmentation into skeleton map, quite often a spurious small segment will be introduced, which is particularly harmful in the context of crossovers. An example is illustrated in Figure 12, where one 4-clique now becomes two 3-cliques, and the geometric properties are also changed, due to the introduction of the spurious segment C which usually is very tiny.

This type of tiny spur can be identified and further removed by checking the average angle *β* and length of the segment *C*: *C* will be removed if *β*≤*β*_*critical*_ and *C*≤*C*_*critical*_. In practice, *β*(*β*_*critical*_)=70° and *C*(*C*_*critical*_)=10.

## Results and discussion

**Figure 13:**
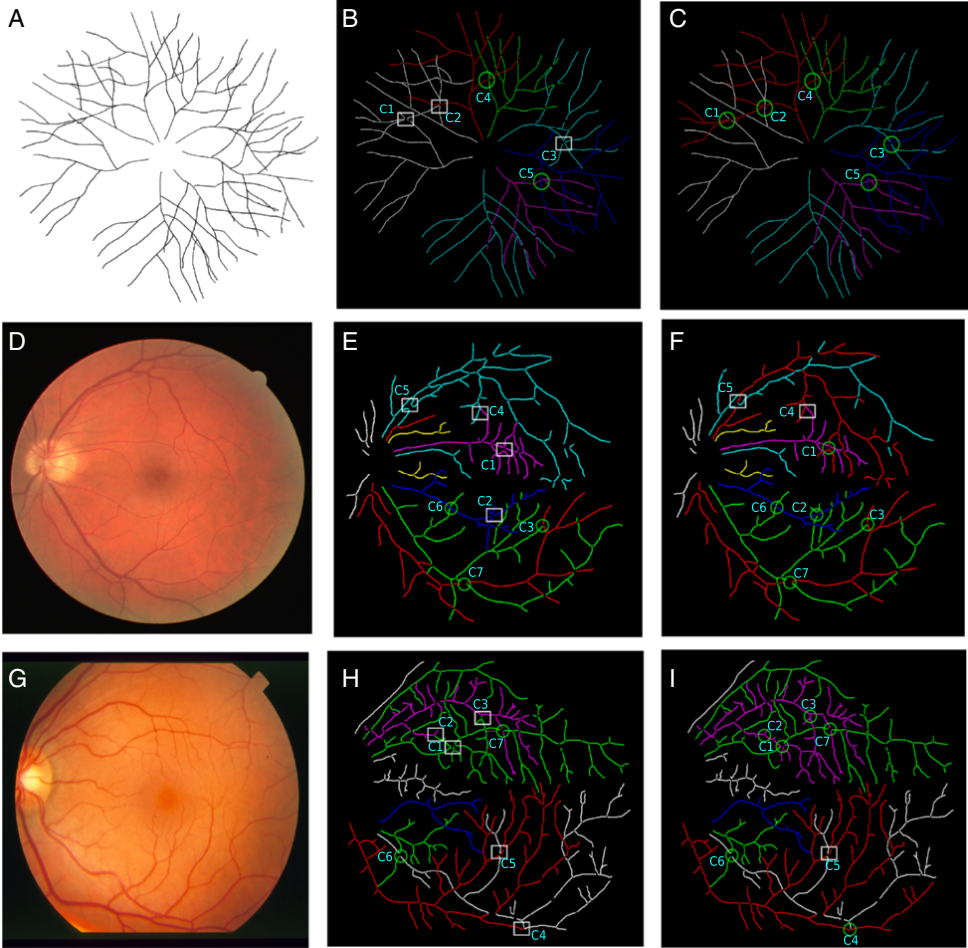
**Tracing results: visual examples.** Visualizing exemplar results for the tracing step. **(A)** Synthetic Image **(B)** Tracing without graph simplification for synthetic data. **(C)** Tracing with graph simplification for synthetic data. **(D)** Image from DRIVE dataset. **(E)** Tracing without graph simplification for DRIVE image. **(F)** Tracing with graph simplification for DRIVE image. **(G)** Image from STARE dataset. **(H)** Tracing without graph simplification for STARE image. **(I)** Tracing with graph simplification for STARE image.

Our approach are evaluated in synthetic datasets, as well as two standard testbeds, DRIVE [31] and STARE [32]. The synthetic datsets contain 17,000 images, while DRIVE dataset contains 40 retinal fundus images, and STARE has 20 fundus images. Some exemplar images of the three types of datasets are shown in Figure 13 subplots (A), (D), and (G). The standard DIADEM score [33] has been used as the evaluation metric.

### Synthetic datasets

**Figure 14:**
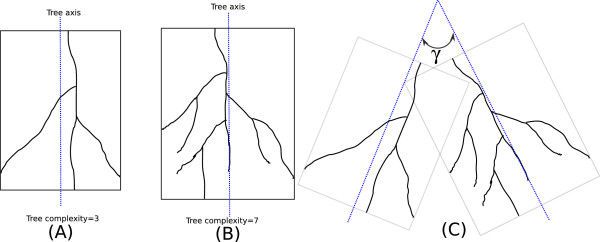
**Illustration on generating synthetic images.(A)** An exemplar tree with low complexity. **(B)** An exemplar tree with medium complexity. **(C)** Illustration on how the synthetic image is generated.

Three synthetic datasets are constructed for systematic performance evaluation under controlled setting, that have in total 17,000 images. The Shreve’s ordering is also used here to quantify the complexity of a tree, measured by the largest ordering value assigned to a tree segment (i.e. the root segment). To start with, thirty tree-like structures are hand-drawn, serving as building-blocks for simulating these synthetic images. These hand-drawn trees can be categorized into three groups: (1) Those with low complexity (root segment ordering value of 2–5); (2) Medium complexity (root value of 5–10); (3) High complexity (root value more than 10). Some examples are presented in Figure 14 subplots (A) and (B), where the tree axis (shown in dotted blue line) is defined in the direction of its length. To generate a synthetic image, the angular distance between two adjacent trees are defined as the spread angle *γ* between the two tree axes in counter-clockwise direction, as shown in subplot (C). Now, a synthetic image can be generated with three parameters: (1) Complexity of the trees in the image; (2) Number of trees; (3) Spread angle. Three synthetic image datasets are thus generated by varying these above parameters, where in each dataset, only one parameter varies while the rest two parameters are fixed.

**Figure 15:**
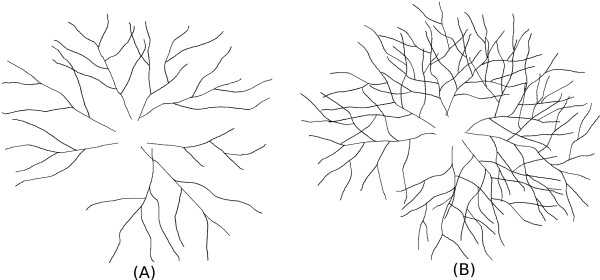
**Tracing: Synthetic dataset 1.** Synthetic Dataset 1: Synthetic images with fixed number of trees and spread angle (angular distance between two adjacent trees), but with varying tree complexity. **(A)** Less complex image. **(B)** Complex image.

**Figure 16:**
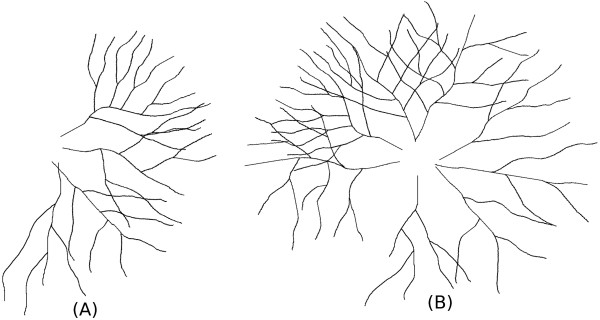
**Tracing: Synthetic dataset 2.** Synthetic Dataset 2: Synthetic images with fixed tree complexity and spread angle, but with varying tree numbers. **(A)**, **(B)** are two synthetic images composed with different number of trees.

**Figure 17:**
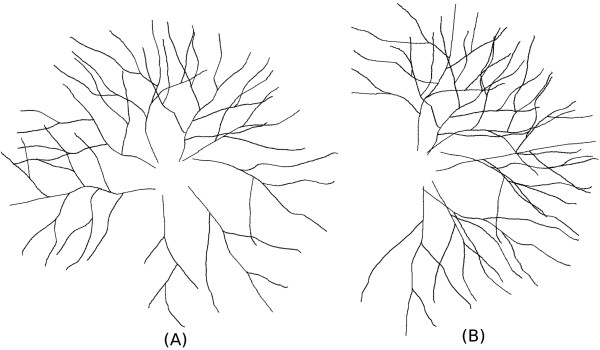
**Tracing: Synthetic dataset 3.** Synthetic Dataset 3: Synthetic images with fixed tree complexity and tree numbers, but with varying spread angle. **(A)**, **(B)** are two synthetic images composed with different spread angles.

• Dataset 1: In this dataset, the tree complexity is varying while the other two parameters are fixed, namely, the number of trees are fixed to 8 and the spread angle is set to 30°. This gives rise to 5 subsets of images within this dataset: (1) All trees are of low complexity; (2) Four out of the eight trees are with low complexity, and the rest four trees are with medium complexity; (3) All trees are of medium complexity; (4) Four out of the eight trees are with medium complexity, and the result four are with high complexity; (5) All trees are of high complexity. Two examples of this dataset are shown in Figure 15. 100 images are produced for each subset, in all 5,000 images are generated in this dataset.

• Dataset 2: In this dataset, the number of trees are varying from the set {3,5,7,9,11,12}, while the other two parameters are fixed: 1/3 from each complexity group, and the angles between trees is set to 30°. Two examples are displayed in Figure 16. Each subset contains 1000 images, in all we have 6,000 images for this dataset.

• Dataset 3: In this dataset, the spread angle varies from the set {360°,300°,240°,180°,120°,60°}, while and the number of trees is set to 8, and for the tree complexity, the same strategy is used as that in dataset 2 (1/3 from each complexity group). Two examples are presented in Figure 17. Each subset contains 1000 images, in all we have 6,000 images for this dataset.

### Preparation for computing the DIADEM score

**Figure 18:**
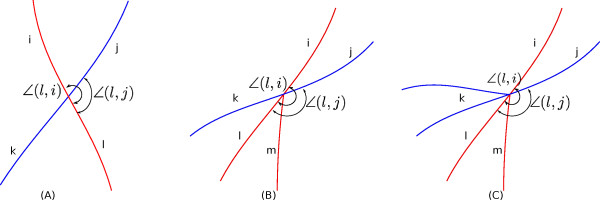
**Preparation prior to computing the DIADEM score.** Preparing the computation of the DIADEM score: Address the self-intersection issues of the tracing results as illustrated in **(A)**, **(B)**, and **(C)**.

DIADEM score operates strictly on tree structures. This holds true for all tracing ground-truth where only trees are presented. Unfortunately our tracing prediction may contain scenarios where branches from the same tree might intersect, and this self-intersection issue need to be resolved before a DIADEM score is computed. The following strategy is proposed to address the issue: Each traced tree is ordered by our modified Shreve’s ordering; We then look at the two segments with largest ordering values (Denoted as (i,j) in the the cases of Figure 18; The (*i*,*j*) segments is thus used to address the self-intersection issue by assigning the rest segments according to angles w.r.t. segments *i* or *j*. For example, in subplot (A), segment *l* will be linked with segment *i*, as *∠*(*l*,*j*) is less than *∠*(*l*,*i*).

### Experimental results

Throughout this paper, the DIADEM score is utilized to measure the performance of a method on a particular dataset, obtained by averaging the scores over all images of current dataset. It has been extensively used in the neural tracing community as standard evaluation metric [33].

### Synthetic datasets

**Figure 19:**
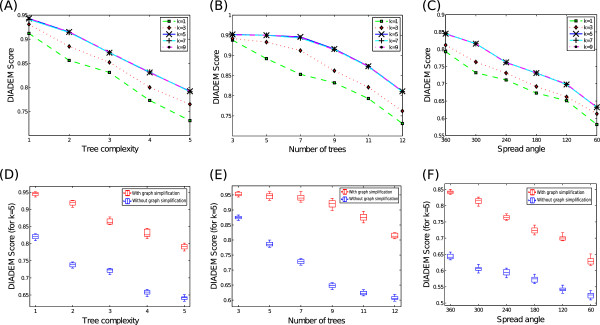
**Tracing results: synthetic experiments.** Performance on synthetic dataset. **(A)** Performance of algorithm with graph simplification with varying *k* for Dataset 1 **(B)** Performance of algorithm with graph simplification with varying *k* for Dataset 2. **(C)** Performance of algorithm with graph simplification with varying *k* for Dataset 3. **(D)** Comparing the performance of algorithm with graph simplification (in red) and without graph simplification (in blue) for Datset 1. **(E)** Comparing the performance of algorithm with graph simplification (in red) and without graph simplification (in blue) for Datset 2. **(F)** Comparing the performance of algorithm with graph simplification (in red) and without graph simplification (in blue) for Datset 3.

The first row of Figure 19, containing three subplots (A)-(C), presents the DIADEM score (for the propose algorithm with graph simplification) for the three synthetic datasets, respectively, where each curve in the plot denotes the DIADEM scores as a function of parameter *k*. Clearly our method is insensitive to a wide range of *k* values (*k*=5,7,9) where they produce almost the same performance, while the score drops significantly by decreasing *k* further to 3 or even 1. Box plots in the second rows of Figure 19, namely subplots (D)-(F) display the corresponding DIADEM score curve with the proposed algorithm with or without the graph simplification module, one for each of the three synthetic datasets. Our complete algorithm (i.e. the one with graph simplification, shown as red boxes) outperforms the one without the graph simplification module by a large (about 10%) margin. The relatively small performance variation around each evaluate point (i.e. each of the box plots) also suggests that our algorithm performs robustly against a variety of inputs.

### Real-life testbeds: DRIVE and STARE

**Figure 20:**
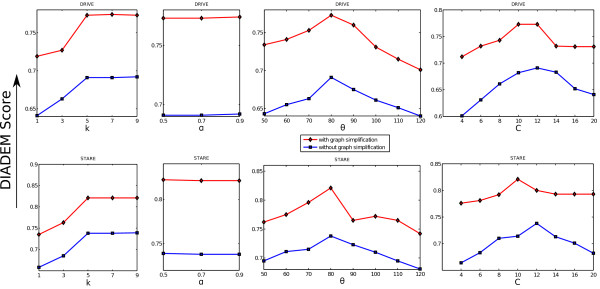
**Tracing results: effect of internal parameters.** The effect on performance by varying internal parameters of the proposed method. The top row denotes the performance for DRIVE dataset and the bottom row denotes the performance for STARE dataset.

In the above mentioned synthetic dataset we have fixed the other parameters and varied only *k* and shown the performance in Figure 20. Another set of experiments are conducted to systematically evaluate the impact of the internal parameters of our algorithm, namely *k*,*α*,*θ*,*C*, on the performance. For each case we fix the rest parameters to their empirically optimum values, while varying the one parameter and plotting the DIADEM scores. This is reported collectively in Figure 20, where the first (second) row of subplots is for DRIVE (STARE) dataset, respectively. During these experiments, we vary *k* in its 5 particular values (1,3,5,7,9), *α* in 3 values (0.5,0.7,0.9), *θ* in 8 values (50°,60°,70°,80°,90°,100°,110°,120°), and *C* in 8 values (4,6,8,10,12,14,16,20). One key observation is the relatively insignificant impacts of varying parameters w.r.t. the final performance, evaluated on real life testbeds. Besides, the changes (if there are any) across the testbeds follow a very similar trend and are usually uni-modal. This facilitate us to pick up a set of empirically optimal set of parameters that we are using throughout this paper, namely, *k*=5, *α*=0.9, *θ*(*θ*_*c*_)80°, and *C*(*C*_*critical*_)=10. It is worth noting that once again our complete algorithm significantly outperform the one without graph simplification module. In the end, our method produces a DIADEM score of 0.765 for DRIVE and 0.821 for STARE, respectively.

The performance of our algorithm is compared with the state-of-the-art tracking based method by Turetken et al. [19] on DRIVE in Table 2. In [19], the authors have varied the cardinality of their k-minimum spanning tree algorithm and plotted the DIADEM scores. For our case we varied each of our 5 internal parameters and plotted five DIADEM score curves in red color (as well as the variation without the graph simplification module as shown in blue) in Figure 20 (top row). In Table 2 we have shown the maximum and minimum DIADEM score obtained by our algorithm and Engin et al. [19]. Our algorithm clearly outperforms the state-of-the-art method, as we have the best DIADEM score of 0.765 which is better than the best DIADEM score of Engin et al. [19]. Besides, the performance of [19] varies dramatically from 0.15 to around 0.71 when varying their internal parameter, which suggests a sense of non-robust behaviour for their system. Meanwhile in our system the performance varies rather mildly, ranging overall from 0.703 to 0.765. Although we have a few internal parameters, our parameters (mostly length and angles) are very intuitive, and their values indeed have only limited impact on the final performance of our work.

**Table 2:** Comparison of DIADEM score (DS) for DRIVE dataset with other methods

**Method**	**Minimum DS**	**Maximum DS**
Our method	.703	.765
Engin et al. [19]	.15	.71

Figure 13 presents the visual results of our work. The first column shows the original image, while the second and the third columns display the corresponding tracing results without and with graph simplification. On the other hand, row number one, two and three denote the tracing results for an exemplar image from the synthetic dataset, the STARE dataset, and the DRIVE dataset, respectively. In the subplot, a white square denotes a wrong tracing result while a green circle denotes a correct result. From subplot (B) we can see that the 5-cliques *C*_1_,*C*_2_ and *C*_3_ are incorrectly traced without the graph simplification module, which become correct when our full model (i.e. with graph simplification) is employed. In both the cases 4-cliques *C*_4_ and *C*_5_ are correctly traced. In subplot (E) for DRIVE, we can see that the 5-cliques *C*_1_ and *C*_2_ are incorrectly traced, while in subplot (F), those are also traced out correctly. Note due to topological errors induced from the segmentation and the skeleton extraction steps, there are a few incorrect tracing results such as *C*_4_,*C*_5_ persist in both subplots (E) and (F) (i.e. without and with graph simplification). Similar patterns also can be observed from the STARE dataset, as shown in the third row (namely subplots (G)-(I)).

## Conclusion

In this paper, we propose a novel approach for tracing retinal blood vessels from fundus images, where we formulate the tracing problem as an equivalent transductive learning problem. Our tracing approach performs very well in resolving many crossover scenarios and various complex situations. It sometimes fails due to imperfect segmentation or in complex scenarios with more than five segments at a junction point. Current results suggest that orientation features are important but might not be sufficient for solving very complex scenarios. As a future direction we are currently working on vessel thickness and texture information for resolving these complex scenarios.

## Endnotes

^1^ More details on transductive inference can be found in Chapter 11 and 24 of [34].

## Appendix

### Details of our segmentation step

To facilitate the tracing step of our pipeline, the goal of the segmentation step here is to extract the vessel skeleton while maintaining the structural connections, as well as the corresponding point-wise radii along the skeleton (A point radius is to measure the thickness of a skeleton point in the orthogonal direction), based on which the retinal vessels can be faithfully reconstructed. This differs notably from the usual aim of most existing segmentation work, where the emphasis is to achieve a high classification accuracy. As the number of vessel pixels are much fewer comparing to the number of background pixels, often a high accuracy is achieved by missing many vessel pixels – a situation we try to avoid. In fact, our goal can be better described as *segmentation with a high recall*. It is critical for us to retain the vessel pixels that keep the local vein and artery branches from being broken or entirely missing. To achieve this, we resort to a cascade of two segmentation modules for producing our final segments. The first one in the cascade is a supervised segmenter as being described next, and the second one is an unsupervised segmenter that is specialized at recovering parallel thin branches, which often tend to be merged into one thick branch by the first module.

#### ***The first segmentation module: supervised segmenter***

In the first module, we implement a supervised segmenter using Gabor filters and GMMs, which is inline with existing supervised methods used for segmenting retinal vessels [6]. For each pixel in the training set, the Gabor response feature of 18 directions are computed and normalized to form the input features [6]. Two GMMs, each having 20 Gaussian components, with one for vessel and the other for non-vessel background pixels, are trained on these features as a pixel classifier. Then for a test image, by applying the trained GMMs we obtain the probability of a pixel being vessel or not. A probability map of the image is produced by maximizing over these two probabilities for each of the pixels.

**Figure 21:**
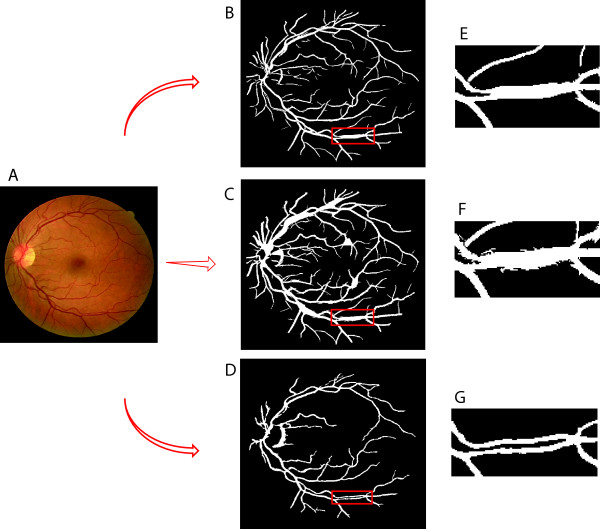
**Our segmenter: Based on existing methods.(A)** Original color fundus image. **(B)** Supervised segmentation result with the highest F1 score. **(C)** Supervised segmentation result with the highest recall. **(D)** Unsupervised segmentation method. **(E)** Two branches are wrongly segmented into one, small branches are disconnected. **(F)** Small branches are often connected back here. **(G)** Two branches are often kept separated from each other.

We have observed that the desired segmentation in our context often stems from the result with highest F1 score, as e.g. demonstrated in Figure 21(B). This is reasonable, since with a high recall (i.e. less false negatives), we are still after a result with high precision (i.e. less false positives). Together they can be characterized by a single F1 score. As demonstrated in Figure 21(B) and (E), the output is reasonably clean, but many small branches of the retinal network are either entirely missing, or not connected. Moreover, the parallel thin branches tend to merge to form a thick branch, which is clearly not desirable for the tracing purpose. Therefore, we further adjust the threshold over a suitable range and obtain a second output with the highest recall: As illustrated in Figure 21(C) and (F), the result tends to be very noisy and much thicker than what it should be. Nevertheless, by starting with the skeleton of the result with highest F1, followed by merging with the small branches from the one with highest recall, we are able to retrieve and reconnect those small branches that otherwise might be missed out. The point-wise radii issue is avoided by simply sticking to the highest F1’s result.

Unfortunately, the result after merging the highest F1 and the highest recall outputs are still not satisfactory: It seems a characteristic of the supervised methods is that they tend to merge very close parallel branches into one branch, undesirable for our purpose of tracing. So we need to consider using a second module from unsupervised segmentation.

#### ***The second module: unsupervised segmenter***

We have attempted with a few existing methods and observed that the segmenter of [8], the one using Isotropic Undecimated Wavelet Transform (IUWT), empirically produces the best segmentation for the close and parallel branches, as illustrated in Figure 21(D) and (G). As a second add-on module of the cascade, based on the current partial result from supervised segmentation, the wrongly-merged thick branches are identified and replaced by the parallel branches from the second module.

#### ***Combining supervised and unsupervised method of segmentation***

For combining the images from supervised and unsupervised method of segmentation (total 3 images) we have followed these steps. 

**Figure 22:**
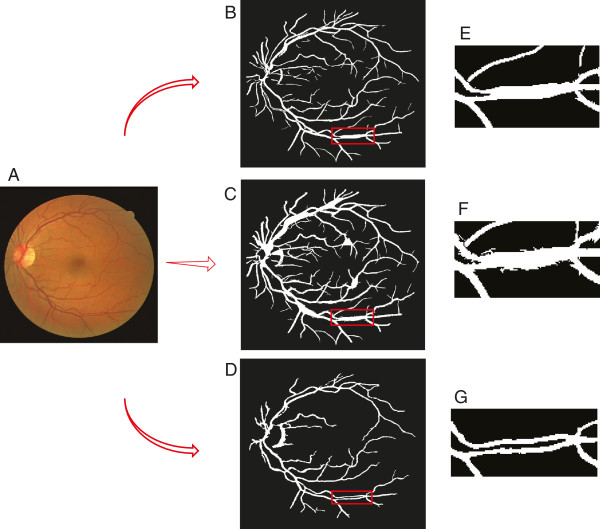
**Our segmenter: Combining the segmentation results.(A)** The partial result so ar, by combining the best elements from Figure 7B-D. **(B)** Final segmentation result. **(C)**-**(D)** Zoom-in views that reveals small branches are still disconnected. **(E)**-**(F)** After the re-connection procedure described in Section ‘Experimental results’, Small branches are now connected.

• We have used the binary segmented images and extracted the skeleton from them.

• Depending on the number of neighbours, we have marked the skeleton pixels as body pixels (those pixels with 2 neighbours), branching pixels (those with 3 or more neighbours) or terminal pixels (those with one neighbour). We define a vessel segment as a group of body pixels which are connected together.

• We calculate the median diameter of each vessel segment by estimating the diameter on each point of the vessel segment skeleton by following the method described in [8]. Then we calculate the mean diameter (*d*_*m*_) of all the vessel segments for a particular image.

• We have replaced the segments which have diameter less *d*_*m*_ from Figure 21-B by the same segments from Figure 21-C. While replacing we always took care about the continuity of the connected segments and we have always preferred thin and longer segments than thick and shorter segments.

• Then we have taken those segments from Figure 22-B, which are one standard deviation more than the *d*_*m*_ and replaced them with the segments from Figure 21-D.

#### ***Resolving the disconnection issue***

So far the partial result is able to retain the small branches, and it works well with the close and parallel branches, as displayed in Figure 22(A). Still some small branches are still disconnected as in Figure 22(C)-(D). The is resolved by first fitting a 3rd-order curve to the skeleton of those disconnected branches, and second, reconnecting them by incrementally and carefully extending a fitted curve from both ends in parallel till it retains contact to a main branch. The radius of each point on the extended curve is estimated as a convex combination of the radii of its neighbouring points, with the weight being in proportion to the inverse distance between the point and its neighbouring point. This finally produces a well-connected structure suitable and ready for tracing purpose (Figure 22(E)-(F)). Note that the segmentation output of an image is represented as the skeleton plus their corresponding point-wise radii.

## Competing interests

The authors declare that they have no competing interests.

## Authors’ contributions

The ideas in this paper are conceived by LC and JD. JD implements and carries out the empirical experiments, while HL contributes important retinal related expertise. Paper is written by all the authors together. All authors read and approved the final manuscript.
